# Nontypable *Haemophilus influenzae* Displays a Prevalent Surface Structure Molecular Pattern in Clinical Isolates

**DOI:** 10.1371/journal.pone.0021133

**Published:** 2011-06-16

**Authors:** Pau Martí-Lliteras, Antonio López-Gómez, Silvia Mauro, Derek W. Hood, Cristina Viadas, Laura Calatayud, Pau Morey, Alain Servin, Josefina Liñares, Antonio Oliver, José Antonio Bengoechea, Junkal Garmendia

**Affiliations:** 1 Programa de Infección e Inmunidad, Fundación Caubet-CIMERA, Bunyola, Spain; 2 Centro de Investigación Biomédica en Red de Enfermedades Respiratorias-CIBERES, Vitoria, Spain; 3 Consejo Superior de Investigaciones Científicas-CSIC, Madrid, Spain; 4 Molecular Infectious Diseases Group, Department of Paediatrics, Weatherall Institute of Molecular Medicine, John Radcliffe Hospital, University of Oxford, Headington, Oxford, United Kingdom; 5 Servicio de Microbiología, Hospital Universitario Bellvitge, Barcelona, Spain; 6 Servicio de Microbiología, Hospital Universitario Son Espases, Palma Mallorca, Spain; 7 Instituto de Agrobiotecnología, CSIC-Universidad Pública de Navarra-Gobierno de Navarra, Mutilva, Spain; 8 Instituto de Investigación Biomédica de Bellvitge (IDIBELL), Barcelona, Spain; 9 Universidad de Barcelona, Barcelona, Spain; 10 INSERM, UMR 756, Signalisation and Physiopathology of Epithelial cells, Paris, France; Indian Institute of Science, India

## Abstract

Non-typable *Haemophilus influenzae* (NTHi) is a Gram negative pathogen that causes acute respiratory infections and is associated with the progression of chronic respiratory diseases. Previous studies have established the existence of a remarkable genetic variability among NTHi strains. In this study we show that, in spite of a high level of genetic heterogeneity, NTHi clinical isolates display a prevalent molecular feature, which could confer fitness during infectious processes. A total of 111 non-isogenic NTHi strains from an identical number of patients, isolated in two distinct geographical locations in the same period of time, were used to analyse nine genes encoding bacterial surface molecules, and revealed the existence of one highly prevalent molecular pattern (*lgtF*+, *lic2A*+, *lic1D*+, *lic3A*+, *lic3B*+, *siaA*−, *lic2C*+, *ompP5*+, *oapA*+) displayed by 94.6% of isolates. Such a genetic profile was associated with a higher bacterial resistance to serum mediated killing and enhanced adherence to human respiratory epithelial cells.

## Introduction

The Gram-negative bacterium nontypable *Haemophilus influenzae* (NTHi) is a commensal organism in the human upper respiratory tract, and a cause of diseases, including otitis media, conjunctivitis, sinusitis, pneumonia, chronic bronchitis and progression of chronic obstructive pulmonary disease (COPD) [1]. This opportunistic pathogen is provided of molecular mechanisms devoted to evade host immunity. A number of studies have reported NTHi ability to resist killing by humoral immunity components such as complement and antimicrobial peptides [2]–[3], and to colonise respiratory epithelial cells by adhesion and biofilm formation [4]–[5], and invasion and intracellular location [6]. Differential genome content between isolates has been reported [7]; this aspect also contributes to bacterial escape from the host immunity. Genetic polymorphisms occur at a high rate within the NTHi population; a major factor contributing to NTHi genetic diversity is DNA exchange by horizontal gene transfer [8]. Phase variation, the reversible high-frequency on/off switching of gene expression mediated by mutations in simple sequence repeats (SSRs) located within the open reading frame (ORF) or the promoter of a gene, also contributes to diversity. SSRs, predominantly comprised of tetranucleotide repeat units, mediate phase variable expression of multiple molecules expressed on the bacterial surface; the repertoire of SSRs varies between NTHi strains [9]. SSRs control expression of epitopes playing a role in molecular mimicry: phosphorylcholine (PCho) mimics the eukaryotic platelet associated factor (PAF) and is a ligand for the PAF receptor; digalactose mimics human P^k^ antigen; sialic acid (5-acetylneuraminic acid or Neu5Ac) is frequently present in host cell membrane glycosphyngolipids [10]–[11].

**Figure 1:**
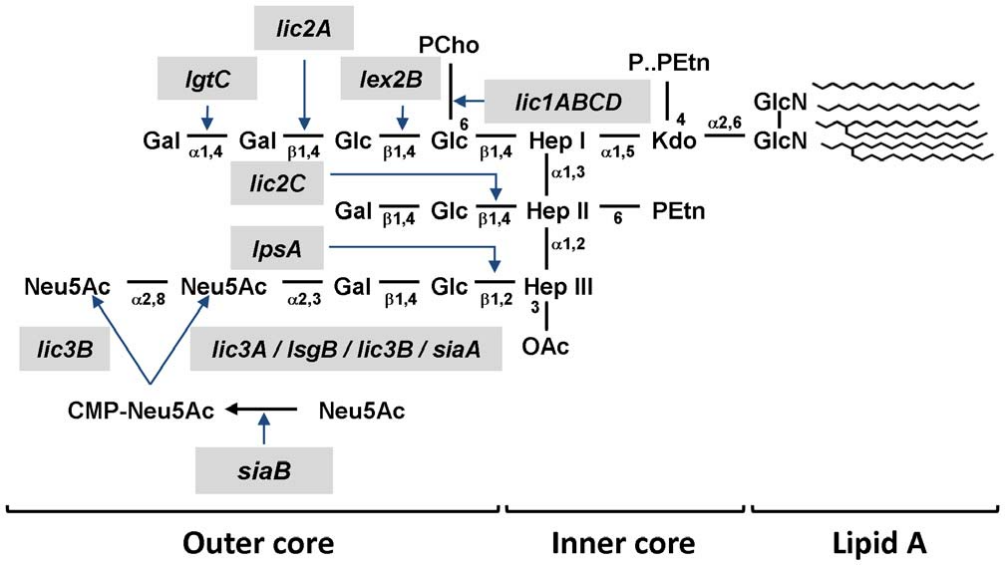
Schematic representation of NTHi LOS structure. Relevant sugars linked by the products from the genes *lgtF*, *lic2C*, *lpsA*, *lic3A*, *lic3B*, *siaA*, *lsgB*, *lic1ABCD*, *lic2A*, *lgtC* and *lex2B* are shown. Abbreviations: Kdo, 2-keto-3-deoxyoctulosonic acid; Hep, L-glycero-D-manno-heptose; Glc, D-glucose; Gal, D-galactose; PEtn, phosphoethanolamine; P, phosphate; PCho, phosphocholine. For each LOS structure the heptose backbone comprises from top to bottom HepI, HepII and HepIII.

Genetic variability between NTHi isolates underlies the striking heterogeneity of the lipooligosaccharide (LOS) outer core, a major virulence determinant. NTHi LOS is a complex glycolipid comprising a membrane-anchoring lipid A linked by a single 2-keto-3-deoxyoctulosonic acid (Kdo) to an heterogeneous oligosaccharide composed of neutral heptose (Hep) and hexose (Hex) sugars, but lacks an O antigen [11]. Each Hep of a conserved trisaccharide inner core can be a point for the addition of Hex and further chain extensions, the degree and pattern of which varies among strains [11] (Fig. 1). The genetic blueprint for NTHi LOS biosynthesis is known; *lgtF* encodes a glycosyltransferase responsible for adding glucose as the first sugar to HepI [12]; *lic2C* and *lpsA* encode glycosyltransferases that initiate sugar extensions from HepII and HepIII, respectively [12]. The *lic1* operon (*lic1A* to *lic1D*) encodes four proteins responsible for the synthesis and transfer of PCho to the LOS [13]. The *lic2A*, *lgtC* and *lex2B* genes encode glycosyltransferases required to add a digalactose to the LOS [14]. Sialic acid is incorporated to LOS as a terminal sugar by four sialyltransferases encoded by *lic3A*, *siaA*, *lsgB* and *lic3B* [15]–[16]. The multiple LOS components contribute to its heterogeneity, and influence the biology of the bacteria [11]. PCho plays a role in NTHi resistance to antimicrobial peptides and serum, association with epithelium, and biofilm formation [3], [17]–[20]. Digalactose is associated with virulence and resistance to complement [21]–[22]. LOS sialylation renders NTHi resistant to complement-mediated killing and plays some role in biofilm formation [23]–[25]. The *lic2C*, *lic2B* and *lic3B* genes are variably present among NTHi strains [12], [15], [24], [26]; *lic1A*, *lic2A*, *lgtC*, *lex2A*, *lic3A* and *lic3B* contain SSRs based on tetranucleotide repeats [9], [27]–[28].

NTHi strains may express a repertoire of proteins to adhere to the respiratory epithelium; typically, PE, Hap, HMW1, HMW2, Hia, Hap, P2, P5 and OapA outer membrane proteins are nonpilus adhesins [29]–[31]. These proteins often display sequence diversity between strains; given their potential variability, two of these proteins were selected for further analysis in the current study. P5, encoded by *ompP5* [32]–[33], is a member of the OmpA protein family; P5 contains host-adhesive domains that bind to human mucin and to the surface-expressed carcinoembryonic antigen-related cell adhesion molecule-1 (CEACAM1) in human pulmonary epithelial cells [34]–[35]. OapA mediates epithelial adhesion via an unknown receptor [29].

A relationship between NTHi pathogenesis and genomic content has been proposed, and several studies have highlighted the importance of LOS and adhesins on NTHi pathogenesis [7], [26], [36]–[41]. In this study, we hypothesised that NTHi isolates could be distinguished by genotypic and/or phenotypic signatures, which could be associated with their capacity to interact with the host. Indeed, a survey of nine genes encoding bacterial surface molecules in a NTHi collection of non-isogenic clinical isolates revealed a highly prevalent molecular pattern with potential clinical implications. Such a molecular pattern could have an influence in bacterial host cell adhesion and in resistance to serum mediated killing.

## Results

### Molecular epidemiology of a collection of NTHi clinical isolates

A total of 111 NTHi strains from 111 patients suffering respiratory (pneumonia, bronchiolitis, sinusitis, acute bronchitis, acute tracheobronchitis, common cold) and non-respiratory (conjunctivitis, bacteremia, otitis media, colonisation in lung carcinoma) infections, and chronic respiratory diseases exacerbations (COPD, cystic fibrosis, bronchiectasis, diffuse parenchymatous lung disease, chronic asthma, chronic interstitial lung disease, polycystic lung) were collected in two Spanish hospitals within January–June 2008 (Table S1). Pulsed field gel electrophoresis (PFGE) analysis indicated that all 111 NTHi isolates had unique genomic compositions (data not shown).

### Distribution and prevalence of bacterial surface structure-related genes

The collection of NTHi isolates was PCR-screened for the presence or absence of nine genes encoding proteins responsible for the expression of surface-exposed molecules (Fig. S1A and S1B and Table S2). Strain Rd KW20 was used as a control because its genome has been fully sequenced [42]. Genes were chosen based on their potential to be variably present (*lic3B*, *siaA*, *lic2C*), variably expressed (*lic2A*, *lic1D*, *lic3A*, *lic3B*), sequence variable (*ompP5*, *oapA*), or invariable present (*lgtF*). The screening revealed three different gene patterns, named 1 to 3 (Table S3). Pattern 2 (*lgtF*+, *lic2A*+, *lic1D*+, *lic3A*+, *lic3B*+, *siaA*−, *lic2C*+, *ompP5*+, *oapA*+) was the most prevalent (94.6% of isolates) in both strain sets (92.2% in Hospital Universitario Bellvitge-HUB, 97.9% in Hospital Son Espases-HSE). NTHi398 [6], [43] belongs to pattern 2. Pattern 1 (*lgtF*+, *lic2A*+, *lic1D*+, *lic3A*+, *lic3B*+, *siaA*−, *lic2C*−, *ompP5*+, *oapA*+), found in 4.5% of isolates, was present only in strains collected in HUB; pattern 3 (*lgtF*+, *lic2A*+, *lic1D*+, *lic3A*+, *lic3B*+, *siaA*+, *lic2C*−, *ompP5*+, *oapA*+), found in 0.9% of isolates, was present only in strains collected in HSE. Main differences among patterns were driven by *lic2C* and *siaA*. Given that those genes encode a glycosyltransferase initiating sugar extension from HepII and a sialyltransferase, respectively, sugar extension from HepII and sialylation were found to be variable modifications in the NTHi LOS molecule.

The *lic2A* gene was present in 100% of strains. Gel electrophoresis revealed *lic2A* size differences among isolates, likely due to the length of the 5′-CAAT-3′ SSR present within the 5′region of *lic2A* ORF (Fig. S1C). Nineteen isolates representing the three molecular patterns defined were selected for further characterization; pattern 1 (NTHi1500, NTHi1559, NTHi1560); pattern 2 (NTHi398, NTHi1513, NTHi1549, NTHi1553, NTHi1556, NTHi1557, NTHi1558, NTHi1566, NTHi1568, NTHi1606, NTHi1607, NTHi1621, NTHi1622, NTHi1623); and pattern 3 (NTHi1619) (Table 1). Those isolates contained between 7 and 33 5′-CAAT-3′ repeats in their *lic2A* coding sequences (Fig. S1C and Table S4). The *lic2A* gene has the 5′-CAAT-3′repeat tract preceded by putative initiation codons in two reading frames, x/y codons in frame 1 and z1/z2 codons in frame 2 [44]–[45]. Fifteen out of 19 (78.9%) isolates contained an in frame *lic2A* gene. The 73.3% of isolates belonging to pattern 2 conserved one reading frame (Table S4). NTHi1550 (pattern 2) showed a *lic2A* PCR product of significantly higher molecular weight than the rest of strains (Fig. S1C), 49 5′-CAAT-3 units in the SSR within this *lic2A* gene, which would be non-permissive for translation.

**Table 1:** Features of NTHi isolates selected for further characterisation.

NTHi strain	Clinical data	Sample type	Molecular pattern	α-PCho reactivity	α-4C4 reactivity	Sialic acid
1500	aCOPD	sputum	1	high	d **−**	e **+**
1559	pneumonia	BAL^f^	1	high	**−**	**−**
1560	COPD	sputum	1	high	**+/−**	**+**
b398	COPD	sputum	2	high	**−**	**+**
1513	COPD	sputum	2	high	**−**	**+**
1549	COPD	transthoracic needle aspiration	2	high	**+/−**	**+**
1553	COPD	sputum	2	double	**−**	**+**
1556	COPD	sputum	2	double	**−**	**+**
1557	COPD	sputum	2	high	**++**	**+**
1558	bronchiectasis	sputum	2	high	**−**	**−**
1566	COPD	sputum	2	double	**−**	**+**
1568	COPD	sputum	2	high	**+/−**	**−**
c1606	bronchiectasis	sputum	2	low	**−**	**+**
1607	COPD	sputum	2	high	**+**	**−**
1621	COPD	bronchial aspiration	2	high	**−**	**−**
1622	bronchiolitis	nasopharynx exudate	2	high	**++**	**+**
1623	COPD	bronchial aspiration	2	high	**−**	**+**
1630	acute tracheobronchiolitis	sputum	2	high	**+++**	**−**
1619	cystic fibrosis	nasopharynx exudate	3	high	**++**	**+**

a COPD: chronic obstructive pulmonary disease.

b 398: reference NTHi strain, isolated in HSE and used previously [36]–[37].

c Strains in this table listed below and including NTHi1606 were isolated in HSE. Strains 1500–1568 were isolated in HUB.

d Signal intensity upon reaction with 4C4 antibody: −, no signal; from +/− to +++, increasing signal.

e LOS sialylation: −, absence of sialylated forms; +, presence of sialylated forms.

f BAL: bronchoalveolar lavage.

The *lic3A* and *lic3B* genes were present in 100% of isolates. PCR products of equivalent size were obtained for each gene in all strains, as the respective SSR was not included in the amplified portion of each gene. The *siaA* gene was found in 0.9% of isolates. The reasons accounting for the low prevalence observed for *siaA* are currently unknown. The extended presence of both *lic3A* and *lic3B* would favour LOS sialylation and, consequently, NTHi phenotypes related to such a LOS modification.

P5 and OapA were selected based on the notion that they display sequence diversity among strains, which could modulate their immunogenic and/or adhesive properties; 100% of isolates contained both genes. The *ompP5* PCR product was size invariable. A PRED-TMBB β-barred analysis predicted five extracellular loop domains (1–5) for P5 [46]. Each predicted P5 extracellular loop was sequenced in the 19 selected isolates (Table S5). A point mutation rendered a premature stop codon in *ompP5* from NTHi1568. Sequence alignments showed different degrees of variability for each predicted loop, i.e. loop 1, 16 different sequences; loop 2, 13 different sequences; loop 3, 11 different sequences; loop 4, 8 different sequences; loop 5, 3 different sequences (Table S5). The sequence of loop 1, predicted to be the longest extracellular domain, was of two types. A region encompassing the motif GINNNGAIK (red) was found in P5 from 17 isolates; two isolates contained the motif GVRAMGKQ (blue). A comparable motif distribution had been reported previously [33]. Loops 2, 3 and 5 were of similar length; loop 4 was the shortest.

The *oapA* PCR products were size variable between strains (data not shown). Six different sequences were found; Table S6 shows sequence differences for the region of the gene starting at aa 195. 61.1% of isolates had Rd KW20-like sequence; 5.6% had Hi Eagan (type b)-like sequence containing a 12 amino acid insertion [29]; other isolates displayed four additional insertion sequences. Taking as a reference the OapA longest sequence, encompassing two QAEQP sequence repeats (red) (NTHi1622), we found 61.1% of strains with a 12 aa deletion, 5.6% of strains with a 32 aa deletion; 5.6% of strains with a 35 aa deletion; 11.1% of strains with a 44 aa deletion; and 11.1% of isolates with a 48 aa deletion (Table S6).

Together, the analysis of seven LOS biosynthesis and two outer membrane protein encoding genes in a collection of NTHi clinical isolates revealed three patterns of distribution, one of them being prevalent.

### NTHi lipooligosaccharide phenotypic distribution among clinical isolates

By having multiple phase-variable loci, NTHi can generate a range of LOS glycoforms that allow the organism to adapt to different host microenvironments [11], [28]. We assessed the expression and distribution of three phase variable LOS modifications involved in virulence, PCho, sialic acid and digalactose, in the subset of NTHi isolates under study. Addition of PCho to NTHi LOS requires the *lic1ABCD* operon [13]. *lic1D*, encoding the PCho transferase, was shown to be present in 100% of strains. PCho incorporation to LOS is dependent on the number of phase-variable 5′-CAAT-3′ repeats in the SSR within the *lic1A* reading frame. All strains expressed PCho; 77.5% of strains (45% from HUB and 32.5% from HSE) displayed high PCho levels; 4.5% displayed low PCho levels (all from HUB); 18% of strains (8.1% from HUB and 9.9% from HSE) displayed a mixture of high or low expressing colonies. 76.2% of strains belonging to the prevalent pattern 2 showed high PCho signal.

**Figure 2:**
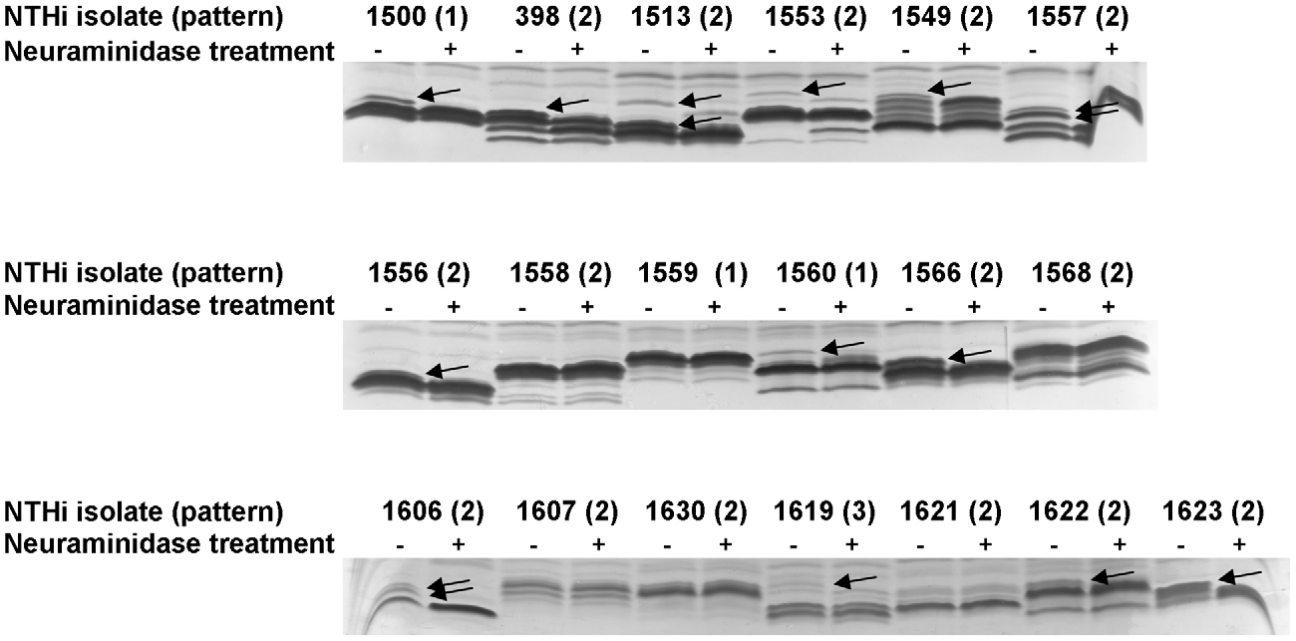
Electrophoresis profiles of LOS isolated from representative NTHi strains. LOS from representative NTHi strains were isolated and separated after SDS-PAGE; strains NTHi1500, 1559, 1560 (pattern 1); NTHi398, 1513, 1549, 1553, 1556, 1557, 1558, 1566, 1568, 1606, 1607, 1621, 1622, 1623, 1630 (pattern 2); NTHi1619 (pattern 3). A pair of profiles, before (−) and after (+) neuraminidase treatment, is shown for each strain. Arrows show bands removed or altered following neuraminidase treatment.

LOS from our selected subset of isolates was analysed by SDS-PAGE, following neuraminidase treatment to assess sialic acid decoration. Electrophoretic profiles showed heterogeneity (Fig. 2). Neuraminidase treatment resulted in lessening or removal of one or more LOS bands in 13/19 (68.4%) of strains, indicating a variable degree and pattern of sialylation (Fig. 2). 66.6% of strains belonging to pattern 2 showed sialylation. All strains contained a combination of two or the three sialyltransferases analysed, *lic3A*, *lic3B* and *siaA*, suggesting that variation in gene content and/or expression could contribute to LOS sialylation diversity.

Analysis of LOS digalactose by dot-blot in our selected subset of isolates showed that 8 of 19 isolates (42.1%) reacted with mAb 4C4; 60% of strains belonging to pattern 2 gave a positive signal with mAb 4C4 (Table 1). Strains with a number of 5′-CAAT-3′ repeats in their *lic2A* SSR non-permissive for translation of full-length Lic2A (see Table S4, NTHi398, 1513, 1556, and 1558) did not react with mAb 4C4. Isolates containing a number of 5′-CAAT-3′ repeats permissive for Lic2A translation displayed heterogeneous 4C4 reactivity (Table 1).

These data showed diversity among NTHi isolates regarding LOS electrophoretic profile that was independent of gene pattern. PCho decorated all LOS analysed and sialic acid was also present in the LOS of most strains tested.

### Contribution of molecular patterns to NTHi serum resistance and epithelial adhesion

We assessed the contribution of the prevalent genetic signature observed to NTHi interaction with the human serum and the respiratory epithelium. We assessed serum mediated killing by incubating bacteria with an increasing range of serum doses (serum percentage ranged between 0.15% and 10%). Table 2 shows serum resistance for three strains belonging to pattern 1 (table 2 upper region, NTHi1500, NTHi1560, NTHi1559), fourteen strains belonging to pattern 2 (table 2 middle region, NTHi1566-NTHi1568), and one strain belonging to pattern 3 (table 2 lower region, NTHi1619). Resistance to serum mediated killing was greater for isolates belonging to pattern 2 (NTHi1566, 1513, 1553, 1557, 398 and 1622) (Table 2). The widest differences among strains were observed at 10, 5 and 2.5% serum. Serum resistance levels correlated to the presence of sialic acid on the LOS (Table 2, labeled with *), in agreement with previous findings [47]. No good correlation between digalactose presence and serum resistance could be established (Table 2, labeled with ^±^).

**Table 2:** Resistance to normal human serum for representative NTHi strains.

NTHi strain			Serum percentage				
	10%	5%	2.5%	1.25%	0.6%	0.3%	0.15%
1500*	0	0	0	1,2	36,8	64,6	89,5
1560* ±	0	8,2	29,2	42,5	60,7	70,8	62,6
1559	1,3	6,7	13,8	23,1	39,1	53,1	68,4
1566*	16	68,6	110,7	102,8	103	102,8	84,6
1513*	13,5	36,5	63,5	82	108,8	105	97,8
1553*	10,9	50	74,1	91,5	94,1	102,1	105,9
1557* ±	0	33,7	55	97,6	98,8	103,6	90,5
398*	0	21,8	79,2	88,4	99,5	93,5	93,7
1622* ±	1,5	10,8	46,4	74,5	77,8	82,5	84,5
1549* ±	2,1	10,8	22,7	38,6	55,9	75,9	81,7
1623*	0,6	5,4	23,2	54,8	75	68,5	82,7
1606*	0	0	36,3	55,5	69,2	76,9	93,4
1607±	2	28,8	59,9	75,4	91,5	94	83,5
1621	0,3	5,4	23,7	63,2	75,3	70,9	77,9
1558	0,3	0,6	14,1	28	48,5	67,6	71,5
1630±	0	0,6	11,4	53,6	80,5	84,7	77
1568±	0	0	0,6	7,2	30,2	56,3	75,8
1619* ±	0,9	4,3	20,1	33,8	53,3	68,5	77,7

Results are expressed as percentage survival of inoculating bacteria against serum concentration.

*LOS is sialylated.

± LOS contains digalactose.

**Figure 3:**
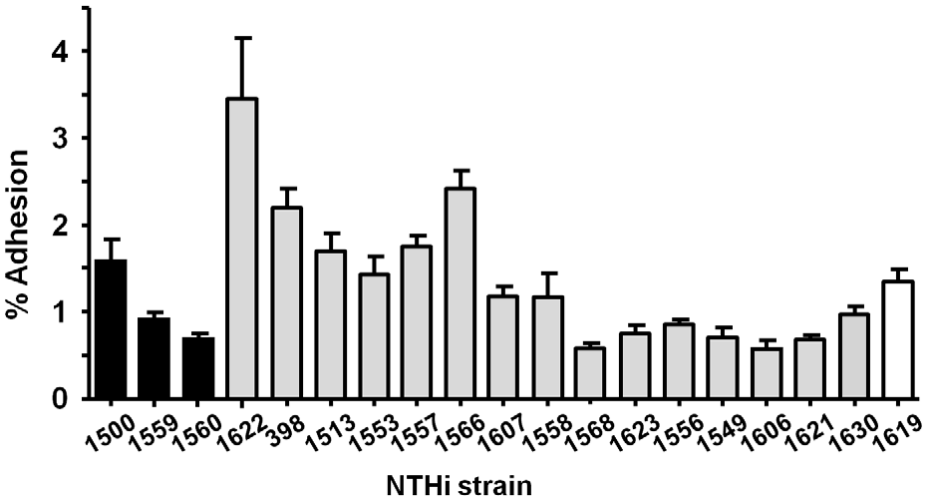
Adhesion of representative NTHi clinical isolates to A549 airway epithelial cells. NTHi strains were used to infect A549 cells at an MOI of 100∶1 for 30 min. Bacterial adhesion was quantified following lysis of host cells, serial dilution, and viable counting. The level of adhesion was determined as follows: percent adhesion = (cfu output/cfu input)×100. Black bars correspond to strains with pattern 1; gray bars to strains with pattern 2; white bar to strain with pattern 3.

We next assessed the adhesion of our selected subset of NTHi strains to human airway epithelium, by using A549 cells as a model. Although the level of bacterial adhesion varied between strains, a number NTHi isolates belonging to pattern 2 (NTHi1622, 398, 1566, 1513, 1557), were more adherent (Fig. 3). NTHi1622 binding to A549 cells was significantly higher than the adhesion of the rest of strains tested; NTHi1566 adhesion was significantly higher than all strains tested, except NTHi398; NTHi398 adhesion was significantly higher than all strains tested, except NTHi1500.

**Figure 4:**
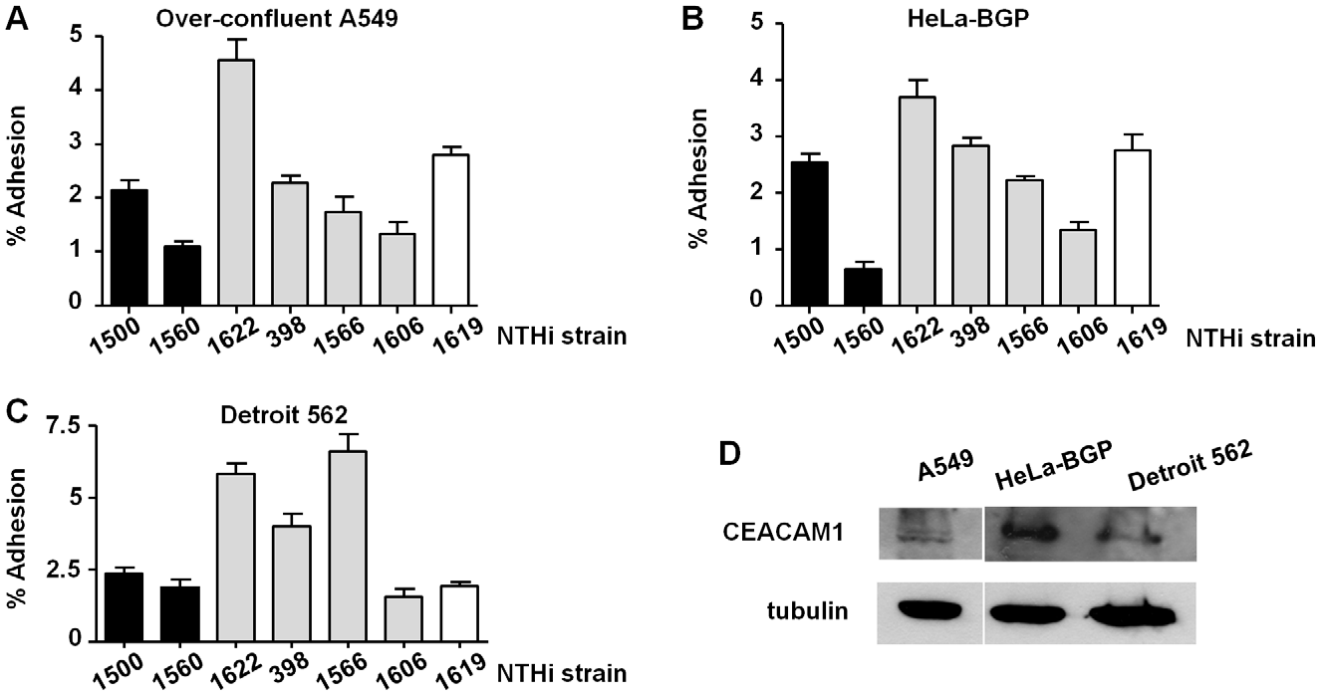
Adhesion of representative NTHi isolates to over-confluent A549, to HeLa-BGP and to Detroit 562 pharynx epithelial cells. NTHi strains were used to infect (**A**) over-confluent A549, (**B**) HeLa-BGP and (**C**) Detroit 562 epithelial cells at an MOI of 100∶1 for 30 min. Bacterial adhesion was quantified following lysis of host cells, serial dilution, and viable counting. The level of adhesion was determined as follows: percent adhesion = (cfu output/cfu input)×100. (**D**) Detection of human CEACAM1 receptor in confluent A549, HeLa-BGP and Detroit 562 epithelial cells by Western Blot with mouse anti-CEACAM1 and goat anti-mouse conjugated to horseradish peroxidase antibodies (upper panel). Extracts were prepared from non-infected cells. Detection of tubulin (bottom panel) was used as control. In (**A**), (**B**) and (**C**), black bars correspond to strains with pattern 1; gray bars to strains with pattern 2; white bar to strain with pattern 3.

The P5 protein has been shown to be a bacterial ligand for the CEACAM1 receptor [34]–[35]. CEACAM1 expression on A549 cells has been reported [35]–[48]; tightly confluent/over-confluent A549 monolayers seem to present significant levels of CEACAM1 [49]. Given that P5 extracellular loops were found to be highly variable among strains, this diversity could account for different bacterial capacity to bind to CEACAM1, which could determine differential bacterial adhesion rates. To guarantee the receptor presence on A549 cells, adhesion assays were carried out by using over-confluent A549 cell monolayers, where CEACAM1 could be detected by western blot (Fig. 4D). Adhesion to A549 over-confluent cells was analysed for seven strains (representative of genetic patterns 1, 2 and 3, and representative of high, medium and low adhesion to A549 cell). Those strains showed a host cell adhesion trend similar to the one shown before (Figs. 3 and 4A). NTHi1622 adhesion was significantly higher than the adherence shown by the rest of strains tested; NTHi1500, 398, 1566 and 1619 displayed an intermediate adhesion capacity; NTHi1560 and NTHi1606 showed lower adhesion than the rest of strains tested.

To further guarantee CEACAM1 presence, identical adhesion assays were carried out by using HeLa-BGP (biliary glycoprotein or CD66a, currently known as CEACAM1) cells [50], a HeLa derivative cell line stably expressing hCEACAM1-4L (Fig. 4D). Adhesion to HeLa-BGP cells was analysed for the same subset of seven strains, displaying host cell adhesion patterns similar to those found for A549 cells (Figs. 3 and 4B). Considering the heterogeneous isolation sources of the NTHi strains under study, we also assessed bacterial adhesion to a different respiratory epithelial cell type. The analysis of NTHi adhesion to Detroit 562 human pharynx epithelial cells for the same subset of seven strains is shown in Fig. 4C. In general terms, NTHi adhesion to Detroit 562 cells was higher than adhesion to A549 or HeLa-BGP cells. NTHi1622 and NTHi1566 displayed higher adhesion than the rest of strains tested. NTHi398 showed an intermediate adhesion level; NTHi1500, 1560, 1606 and 1619 seemed to be lower binders. CEACAM1 could also be detected on Detroit 562 cell extracts (Fig. 4D).

In summary, NTHi isolates belonging to the most prevalent gene pattern are more resistant to complement mediated killing and adhere better to airway epithelial cells. Adhesion capacity varies among strains. Comparable adhesion diversity was found for a subset of strains showing different P5 extracellular loops sequence when adhesion to three independent human cell lines expressing CEACAM1 receptor was assessed.

## Discussion

We analysed the distribution, conservation and expression of genes encoding NTHi surface structures, which revealed a prevalent molecular pattern in a collection of non-isogenic NTHi clinical isolates from different pathological origins. The fact that all isolates rendered different PFGE profiles gives further support to the notion that NTHi genetic content is heterogeneous among isolates [8]. Strains belonging to the identified prevalent pattern showed prominent levels of resistance to serum mediated killing and adhesion to respiratory epithelium, suggesting that this molecular signature could be advantageous for NTHi pathogenicity. This notion has been observed for *Staphylococcus aureus*, by establishing genetic relationships between nasal carriage and clinical isolates [51]. Strains endowed with the identified prevalent signature contain *lgtF*, *lic2A*, *lic1D*, *lic3A*, *lic3B*, *lic2C*, *ompP5* and *oapA*. Of note, Rd KW20, extensively used as a reference strain in *H. influenzae* studies, does not present the same molecular profile. Indeed, Rd KW20 molecular profile is *lgtF*+, *lic2A*+, *lic1D*+, *lic3A*+, *lic3B*−, *siaA*+, *lic2C*−, *ompP5*+, *oapA*+. Gene pattern 2 facilitates sugar chain extensions from HepI and HepII in the LOS. Although sugar extensions from HepIII were not analysed here, *lpsA* is present in NTHi strains tested to date [12]. A previous survey on NTHi otitis media isolates showed *lic2C* in 48.1% of strains [12]. We observed significantly higher *lic2C* prevalence (94.6%) in our NTHi collection. Same trend was observed for *lic3B*, present in 100% of isolates in our collection, but in 60% of previously analysed NTHi otitis media isolates [15]. Such different gene prevalence could relate to differences in strain collection size or anatomical origin.

The *lic1A*, *lic2A*, *lic3A* and *lic3B* genes contain multiple copies of a tetranucleotide repeat within the 5′end of their coding sequences. The repeat copy number can vary due to slipped-strand mispairing, resulting in altered gene expression and LOS structural variation [28]. Our complete strain collection expressed PCho, mostly at a high level. Although PCho addition to LOS is variable, dependent on *lic1A* phase variation [22], its ubiquitous expression in our isolates would support that PCho expression is advantageous for the pathogen. In fact, PCho promotes NTHi infection and persistence by reducing host inflammatory response and promoting the formation of stable biofilms [17]–[18], [52]. The *lic2A* gene was also present in all isolates; however, when LOS decoration with digalactose was assessed, expression was variable within and between strains. MAb 4C4 interacts with a digalactoside epitope when located as the terminus of a globotetraose extension from HepI or HepII; this expression could change due to phase variation in the *lic2A*, *lgtC* or *lex2* loci. All isolates tested carry at least two sialyltransferase genes and most isolates express sialylated LOS. Four sialyltransferase encoding genes have been identified so far in *H. influenzae* strains [15]–[16]. This functional redundancy could be focused to favor LOS sialylation, supporting the notion of a relevant role for this LOS modification during NTHi infection. In agreement with previous reports [24], we found a correlation between LOS sialylation and serum resistance. Of note, epidemiological data suggest that LOS sialylation could be linked to disease severity [53].

The *ompP5* gene encodes putative surface-exposed loops, highly heterogeneous between strains. Previous studies showed *ompP5* sequence variability [32]–[33]; an *ompP5* orthologue in *H. parasuis* shows diversity among isolates [46]. Given that P5 has immunodominant properties [54], NTHi is likely to evolve a high variability rate in this protein to evade adaptive immunity. P5 also displays host cell adhesive properties, by binding to CEACAM1 receptor [34]–[35]; P5 observed diversity may modulate bacterial adhesion to cell surfaces, which may be related to the adhesion heterogeneity among the NTHi strains analysed in this study. NTHi adhesion to A549 and HeLa-BGP cells was similar for the strains tested; NTHi adhesion to Detroit 562 cells was comparable, in general terms, to adhesion to A549 and HeLa-BGP cells, with subtle differences. Of note, different cell types are endowed with distinct receptor molecules; thus, adhesion variability between cell types could be related not necessarily to P5-CEACAM1 adhesion dynamics, but to additional bacterial adhesive molecules which would bind their partners on the host cell surface. OapA is an envelope protein contributing to nasopharynx colonization [31] and cell adhesion [29]. OapA sequence diversity observed here may modulate bacterial interaction with cell surfaces. Adhesion is generally a crucial stage during infection processes [55]. NTHi adhesion is the result of the combined effect of multiple variable adhesive molecules, the combinations of which vary among strains. Strains tested here displayed adhesion heterogeneity; even though, the most adherent isolates belonged to the most prevalent molecular pattern. Of note, NTHi1622, NTHi398 and NTHi1566, displaying significantly higher adhesion rates, contained long OapA sequences, the prevalent GINNNGAIK motif in their P5 loop 1, and PCho and sialic acid decorating their LOS.

Bacterial population studies have suggested the association of the genotype *IS1016*+, *hia*+, *hmw*− with NTHi invasive isolates [41], the presence of the *IS1016-bexA* deletion with non-type b *H. influenzae* [39], and the association of *lic2B*+, *hmw*+ and nine other genomic loci with NTHi otitis media isolates [37]. We present here a molecular profile found prevalent in a collection of NTHi strains of heterogeneous clinical origin. Based on our observations, we postulate that this profile could be a general feature providing the pathogen with advantages during infectious processes. These findings provide information with potential clinical implication. The prevalent bacterial surface structures could be suitable targets for designing new antimicrobial molecules. Future studies will attempt to expand our knowledge about the features dependent upon the molecular signature presented here.

## Materials and Methods

### Ethics statement for collection of NTHi clinical isolates

The study was approved by the Ethics Committee of the Balearic Islands and all aspects of the study comply with the Declaration of Helsinki. Ethics Committee of the Balearic Islands specifically approved that not informed consent was required because data were going to be analysed anonymously. Bacterial strains were isolated from human clinical samples which were collected following Hospital Universitario Bellvitge and Hospital Universitario Son Espases approved procedures by each Microbiology Service.

### Bacterial strains and culture conditions

NTHi strains from 111 patients from 17 days to 92 years of age were isolated between January and June 2008 in Hospital Bellvitge-HUB (Barcelona) and Hospital Son Espases-HSE (Mallorca), two Spanish tertiary reference hospitals. Sixty four strains were isolated in HUB from sputum (52 strains), bronchial or tracheal aspirate (5), conjunctive exudate (2), blood (2), bronchoalveolar lavage (BAL) (2) and pulmonary abcess (1). Forty seven strains were isolated in HSE from sputum (24), bronchial or tracheal aspirate (14), nasopharyngeal exudate (5), conjunctive exudate (3) and ear exudate (1). Rd KW20 is a non-capsular serotype d-derived strain for which a genome sequence has been completed [42]. NTHi398 is a COPD isolate [6], [43]. NTHi was grown at 37°C with 5%CO_2_ on Chocolate-agar plates or on Brain Heart Infusion (BHI)-agar supplemented with hemin (10 µg/ml) and β-NAD (β-nicotinamide adenine dinucleotide, 2 µg/ml).

### Pulse field gel electrophoresis (PFGE)

NTHi isolates were characterised by macrorestriction analysis of chromosomal DNA after *Sma*I digestion and fragment separation by PFGE using a CHEF-DR III contour-clamped homogeneous electric field apparatus (BioRad), programmed at 6 V/cm^2^ for 23 h, with switching times ramped from 1 to 30 s. DNA fragments were visualised by ethidium bromide staining and interpreted following previous criteria [56].

### Genomic DNA preparation and PCR amplification

Chromosomal DNA was isolated from a 5 ml culture grown overnight in sBHI. Bacteria were centrifuged (14.000 g, 5 min), the pellet was resuspended in 567 µl of TE. Subsequently, 30 µl of 10% SDS and 3 µl of proteinase K (20 mg/ml) were added, mixed and incubated, 1 h at 37°C. Then, 100 µl of 5 M NaCl were added, followed by 80 µl of 10% CTAB/0.7 M NaCl. Samples were incubated, 65°C for 10 min. An equivalent volume of chloroform/isoamyl alcohol was added, mixed and centrifuged (14.000 g, 5 min). The aqueous supernatant was transferred to a fresh tube, and re-extracted with phenol/chloroform/isoamyl alcohol. Thereafter, 0.6 ml of isopropanol were added to the supernatant, the mixture was centrifuged. The pellet was washed twice with 70% ethanol, dried, and resuspended in 100 µl sterile water.

Genes were screened with primers in Table S2: *lgtF* (lgtF-F1+lgtF-R1, 1461 bp), *lic2A* (lic2A-Fs+lic2A-Rs, 909 bp), *lic1D* (lic1D-F1+lic1D-R1, 826 bp), *lic3A* (lic3A-F1+lic3A-R1, 888 bp; lic3A-F2+lic3A-R1, 846 bp), *lic3B* (lic3B-F1+lic3B-R1, 891 bp; lic3B-F2+lic3B-R1, 851 bp), *siaA* (siaA-F1+siaA-R1, 854 bp), *lic2C* (lic2BA+lic2BC, 1035 bp; lic2BA+lic2BB, 1898 bp), *ompP5* (P5-Fw+P5-Rv, 1046 bp; P5-seqF4+P5-Rv, 912 bp; P5-seqF5+P5-Rv, 985 bp), *oapA* (oapA-Fw+oapA-Rv, 1170 bp). PCR was performed on chromosomal DNA using Taq DNA polymerase: 5 min denaturation (94°C); 1 min periods of denaturation (94°C), annealing (55°C) and polymerisation (72°C) for 30 cycles. For genes where one single product size was observed for all isolates, PCR products from two randomly chosen isolates were sequenced. For *ompP5*, PCR products from the subset of selected strains were sequenced. For *lic2A* and *oapA*, at least one representative PCR product per size was sequenced.

### Colony immunoblot

PCho level was assessed by immunoblotting as described previously, by using mouse anti-PCho TEPC-15 antibody [13].

### Analysis of LPS by electrophoresis and dot-blot

LPS patterns from selected NTHi isolates were analysed as previously described [57]. Bacterial lysates were prepared from cells grown overnight on BHI plates supplemented with Neu5Ac, then suspended in PBS (pH 5.8) to a concentration with an absorbance of 1 at 260 nm. Lysates were treated with neuraminidase and analysed by Tricine-SDS-PAGE and staining with silver (Quicksilver; Amersham Biosciences). The neuraminidase, purified from *Clostridium perfringens*, cleaves terminal sialic acids bound to oligosaccharides. Assessment of digalactose levels on the surface of NTHi strains was performed using 2 µl lots of bacterial lysates in dot-blots [57], using monoclonal antibody 4C4, which recognises Gal-α-(1-4)-β-Gal [58].

### Cell culture and bacterial infection

A549 human alveolar epithelium (immortalised type II pneumocytes, ATCC CCL-185) was maintained as before [6]. Cells were seeded (to reach 4×10^5^ cells/well) in 24-well plates, and serum starved 16 h before infection by replacement of medium with supplemented RPMI lacking FBS. When necessary, cells were seeded for a longer period of time to reach an over-confluent monolayer (approximately 8×10^5^–1×10^6^ cells/well) [49]. HeLa-BGP is a stably transfected HeLa cell line expressing hCEACAM1-4L receptor [50]. HeLa-BGP cell line was propagated as described before [50]. Cells were seeded (4×10^5^ cells/well) in 24-well plates, 24 h before each experiment. Detroit 562 human pharynx epithelium (immortalised pharynx epithelial cell line, ATCC CCL-138) was maintained following manufacture's instructions. Briefly, Detroit 562 cells were maintained in DMEM tissue culture medium supplemented with 1% HEPES, 10% heat inactivated foetal calf serum (FCS) and antibiotics (penicillin and streptomycin) in 25 cm^2^ tissue culture flasks at 37°C in a humified 5% CO_2_ atmosphere. Cells were seeded (10^5^ cells/well) in 24-well plates, 24 h before each experiment. Stationary phase grown NTHi were recovered with 1 ml PBS from a Chocolate-agar plate and adjusted to OD_600_ = 1 (10^9^ c.f.u./ml). In all cases, cells were infected in 1 ml EBSS (Earle's Balanced Salt Solution) with the freshly obtained bacterial suspension, at approximately MOI 100∶1, for 30 min, washed three times with PBS, and lysed with 300 µl of PBS-Saponin 0.025% for 10 min at room temperature. Serial dilutions were plated on sBHI-agar. Bacterial adhesion is represented as % = (c.f.u. output/c.f.u. input)×100.

### Serum resistance assay

Bacteria grown on medium supplemented with Neu5Ac (30 µg/ml) were assayed for survival against the killing effect of normal human serum [24].

### Detection of CEACAM1 by Western blotting

A549 cells were seeded on 6-well tissue culture plates to reach over-confluence (approximately 4×10^6^ cells/well). HeLa-BGP cells were seeded on 6-well tissue culture plates at 2×10^6^ cells/well. Detroit 562 cells were seeded on 21 cm^2^ tissue culture dishes at 10^7^ cells/dish. In all cases, cells were highly confluent before processing. Cells were washed 3 times with cold PBS, scraped and lysed with 100 µl lysis buffer (1× SDS Sample Buffer, 62.5 mM Tris-HCl pH 6.8, 2% w/v SDS, 10% glycerol, 50 mM DTT, 0.01% w/v bromophenol blue) on ice. Samples were sonicated, boiled at 100°C for 10 min and cooled on ice before polyacrylamide gel electrophoresis and Western Blotting. CEACAM1 was detected with primary mouse anti-human CEACAM1 (R&D Systems, clone MAB2244) antibody diluted 1∶500 and secondary goat anti-mouse antibody conjugated to horseradish peroxidase (Thermo Scientific) diluted 1∶1,000. Tubulin was detected with primary mouse anti-tubulin antibody (Sigma) diluted 1∶3,000 and secondary goat anti-mouse antibody (Pierce) conjugated to horseradish peroxidase diluted 1∶1,000. When necessary, the membrane was washed twice for 15 min with PBS-0.5% Tween-20, incubated for 30 min in Restore™ Western Blot Stripping Buffer (Thermo Scientific) at 37°C, and washed twice for 15 min with PBS-0.5% Tween-20.

### Statistical methods

Statistical analyses were performed using analysis of variance, the two sample *t* test with two tails followed by the Mann Whitney test. When required, ANOVA followed by Bonferroni's multiple comparison test was performed. A P value of <0.05 was considered statistically significant. Analyses were performed using GraphPad.

## Supporting Information

Figure S1 **Location of primers and PCR fragment sizes of genes analysed in the present study.** (**A**) Localization of primers used for PCR amplification of LOS biosynthesis and adhesin encoding genes in a collection of NTHi clinical strains. (**B**) 1% agarose gel showing representative PCR products obtained for *lgtF*, *lic2C*, *lic1D*, *lic2A*, *lic3A*, *lic3B*, *siaA*, *ompP5* and *oapA*. (**C**) 1% agarose gel showing representative *lic2A* PCR products displaying size differences associated with variations in the number of -CAAT- repeats in the 5′region of the reading frame. (-CAAT-)_x_ refers to the number of tetranucleotide repeats present in strains Rd KW20 (22 repeats), NTHi1609 (10 repeats), NTHi1500 (11 repeats), NTHi1525 (15 repeats), NTHi1556 (18 repeats), NTHi1549 (20 repeats), NTHi1501 (26 repeats), NTHi1553 (31 repeats), NTHi1550 (49 repeats). 1 Kb and 100 bp ladders (New England Biolabs) were used to asses PCR product sizes (**B** and **C**). 1 Kb ladder includes fragments of 10, 8, 6, 5, 4, 3, 2, 1.5, 1 and 0.5 Kb. 100 bp ladder includes fragments of 1.517; 1.2; 1 (Kb), 900, 800, 700, 600, 500, 400, 300, 200 and 100 bp.
(TIF)

Table S1 **Clinical origin of NTHi isolates used in this study.**
(DOC)

Table S2 **Primers used in this study.**
(DOC)

Table S3 **Distribution of LOS biosynthesis genes in a collection of 111 clinical NTHi isolates.**
(DOC)

Table S4 **Distribution of** ***lic2A*** **phase-variable 5′-CAAT-3′tetranucleotide repeats for representative NTHi isolates.**
(DOC)

Table S5 **P5 sequence in predicted extracellular loops 1 to 5 for representative NTHi isolates.**
(DOC)

Table S6 **OapA protein sequence in the variable region identified for representative NTHi isolates.**
(DOC)
