# Supplementary figures and images for: Nontypable Haemophilus influenzae Displays a Prevalent Surface Structure Molecular Pattern in Clinical Isolates
Source: PLoS One. 2011 Jun 16;6(6):e21133. doi: 10.1371/journal.pone.0021133 (PMC3116884; doi:10.1371/journal.pone.0021133)

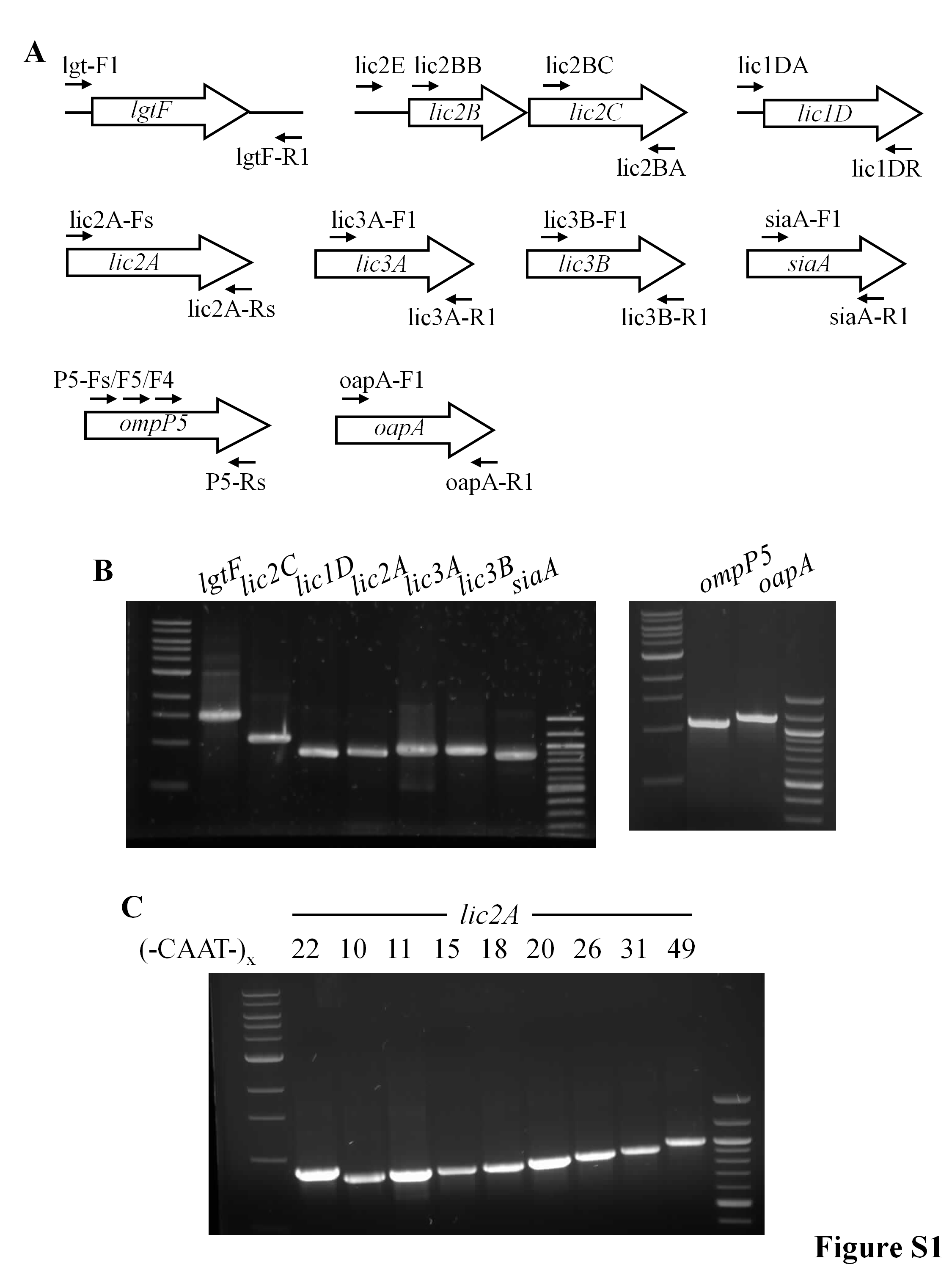

Supplement: Figure S1 — Location of primers and PCR fragment sizes of genes analysed in the present study. (A) Localization of primers used for PCR amplification of LOS biosynthesis and adhesin encoding genes in a collection of NTHi clinical strains. (B) 1% agarose gel showing representative PCR products obtained for lgtF, lic2C, lic1D, lic2A, lic3A, lic3B, siaA, ompP5 and oapA. (C) 1% agarose gel showing representative lic2A PCR products displaying size differences associated with variations in the number of -CAAT- repeats in the 5′region of the reading frame. (-CAAT-)x refers to the number of tetranucleotide repeats present in strains Rd KW20 (22 repeats), NTHi1609 (10 repeats), NTHi1500 (11 repeats), NTHi1525 (15 repeats), NTHi1556 (18 repeats), NTHi1549 (20 repeats), NTHi1501 (26 repeats), NTHi1553 (31 repeats), NTHi1550 (49 repeats). 1 Kb and 100 bp ladders (New England Biolabs) were used to asses PCR product sizes (B and C). 1 Kb ladder includes fragments of 10, 8, 6, 5, 4, 3, 2, 1.5, 1 and 0.5 Kb. 100 bp ladder includes fragments of 1.517; 1.2; 1 (Kb), 900, 800, 700, 600, 500, 400, 300, 200 and 100 bp. (TIF) [file pone.0021133.s001.tif]
