# Supplementary material for: Nontypable Haemophilus influenzae Displays a Prevalent Surface Structure Molecular Pattern in Clinical Isolates
Source: PLoS One. 2011 Jun 16;6(6):e21133. doi: 10.1371/journal.pone.0021133 (PMC3116884; doi:10.1371/journal.pone.0021133)
Supplement: Table S1 — Clinical origin of NTHi isolates used in this study. (DOC) [file pone.0021133.s002.doc]

# Table S1. Clinical origin of NTHi isolates used in this study.

| **Clinical data** | **Nº strains (HUBellvitge-HUB**  **/HSEspases-HSE)** |
| --- | --- |
| **COPD** | **47 (30/17)** |
| **Other chronic respiratory diseases** | **15 (7/8)** |
| Cystic fibrosis | 6 (0/6) |
| Bronchiectasis | 4 (3/1) |
| Diffuse parenchymatous lung disease | 2 (1/1) |
| Chronic asthma | 1 (1/0) |
| Chronic interstitial lung disease | 1 (1/0) |
| Polycystic lung | 1 (1/0) |
| **Respiratory infections** | **36 (20/16)** |
| Pneumonia | 14 (6/8) |
| Bronchiolitis | 1 (0/1) |
| Sinusitis | 1 (1/0) |
| Acute bronchitis | 17 (12/5) |
| Acute tracheobronchitis | 2 (0/2) |
| Common cold | 1 (1/0) |
| **Non-respiratory infections** | **10 (5/5)** |
| Conjuntivitis | 4 (2/2) |
| Bacteremia | 1 (1/0) |
| Otitis media | 2 (0/2) |
| Colonization in lung carcinoma | 3 (2/1) |
| **Data not-accessible** | **3 (2/1)** |
