# Supplementary material for: Nontypable Haemophilus influenzae Displays a Prevalent Surface Structure Molecular Pattern in Clinical Isolates
Source: PLoS One. 2011 Jun 16;6(6):e21133. doi: 10.1371/journal.pone.0021133 (PMC3116884; doi:10.1371/journal.pone.0021133)
Supplement: Table S2 — Primers used in this study. (DOC) [file pone.0021133.s003.doc]

**Table S2**. Primers used in this study.

| **Name** | **5´-3´sequence** | **Reference** |
| --- | --- | --- |
| lgtF-F1 | TGGTGGTGGGCAAGACGC | [7] |
| lgtF-R1 | AGCCTGAATTCGACAGCC | [7] |
| lic2BC | CAATTTCACTAACTTGCC | [7] |
| lic2BA  lic2BB | CAATTTAGCGATGAGTTCC  AAGTATGATCCTCAAATG | [7] |
| lic1DA | GTGATGATATATTTGAAATG | [39] |
| lic1DR | TGGAAGCTTCATATAATCTCCATAA | [39] |
| lic2A-Fs | ATGAGTGCTATTGAAAATATTGTCATT | This study |
| lic2A-Rs | CTACATAAAACGAACAATTTCTTTACCTTG | This study |
| lic3A-F1  lic3A-F2 | CTTAGGAGGTCATATGTCAAAGTCTGTCATTATTGCAGGTAATGG  TCAATTGACTATAGTTTATTACCTAAA | [10]  This study |
| lic3A-R1 | CCTAGGTCGACCTAATCCCATTTTCTTGATTTTAAGGCGTG | [10] |
| lic3B-F1  lic3B-F2 | CTTAGGAGGTCATATGTCAAAGCCTGTCATTATTGCAGGTAATGG  AATCAATTGACTATAGTTTATTACCTA | [10]  This study |
| lic3B-R1 | CCTAGGTCGACTTATTTGCGTAGTCTCATTTTCTTTGC | [10] |
| siaA-F1 | GATGTTATTTTTATTTTTGTT | This study |
| siaA-R1 | ACTTAGGGTGTATTTTGGTTCC | This study |
| oapA-Fw | CAGCCAAGTTCGTCAATTTTAGGTAAAGCA | This study |
| oapA-Rv | CACAAAACGCGCACCATTGGATAAACGCAT | This study |
| P5-Fw | CTGCAATCGCATTAGTAGTTGCTGGTTTAG | This study |
| P5-Rv  P5-seqF4  P5-seqF5 | AGTACCGTTTACCGCGATTTCTACACGACG  GAATATAAAGTAGGTTATCACCGTAATTCT  GAAAACACTTTCTACGCTGGCGTTAAAGCT | This study  This study  This study |
