# Supplementary material for: Nontypable Haemophilus influenzae Displays a Prevalent Surface Structure Molecular Pattern in Clinical Isolates
Source: PLoS One. 2011 Jun 16;6(6):e21133. doi: 10.1371/journal.pone.0021133 (PMC3116884; doi:10.1371/journal.pone.0021133)
Supplement: Table S3 — Distribution of LOS biosynthesis genes in a collection of 111 clinical NTHi isolates. (DOC) [file pone.0021133.s004.doc]

**Table S3. Distribution of LOS biosynthesis genes in a collection of 111 clinical NTHi isolates.**

| **Pattern** | *lgtF* | *lic2A* | *lic1D* | *lic3A* | *lic3B* | *siaA* | *lic2C* | *ompP5* | *oapA* | Total nº  (HUB/HSE) | % (HUB/HSE) |
| --- | --- | --- | --- | --- | --- | --- | --- | --- | --- | --- | --- |
| **1** | + | + | + | + | + | - | - | + | + | 5 (5/0) | (4.5/0) |
| **2** | + | + | + | + | + | - | + | + | + | 105 (59/46) | 94.6 (92.2/97.9) |
| **3** | + | + | + | + | + | + | - | + | + | 1 (0/1) | 0.9 (0/2.1) |
