# Supplementary material for: Nontypable Haemophilus influenzae Displays a Prevalent Surface Structure Molecular Pattern in Clinical Isolates
Source: PLoS One. 2011 Jun 16;6(6):e21133. doi: 10.1371/journal.pone.0021133 (PMC3116884; doi:10.1371/journal.pone.0021133)
Supplement: Table S4 — Distribution of lic2A phase-variable 5′-CAAT-3′tetranucleotide repeats for representative NTHi isolates. (DOC) [file pone.0021133.s005.doc]

**Table S4.** Distribution of *lic2A* phase-variable 5´-CAAT-3´tetranucleotide repeats for representative NTHi isolates.

| **NTHi strain (Pattern)** | **Sequence** | **Lic2A translation** |
| --- | --- | --- |
| **Rd KW20** | Frame x/y: ATG_x_-ATG_y_-(CAAT)_22_-CA AAC TCC | yes |
|  | Frame z1/z2: ATG_z1_-ATG_z2_-(CAAT)_22_-CAA ACT CC | no |
| **NTHi1560 (1)** | Frame x/y: ATG_x_-ATG_y_-(CAAT)_7_-CA AAC TCC | yes |
|  | Frame z1/z2: ATG_z1_-ATG_z2_-(CAAT)_7_-CAA ACT CC | no |
| **NTHi1559 (1)/1630 (2)** | Frame x/y: ATG_x_-ATG_y_-(CAAT)_10_-CA AAC TCC Frame z1/z2: ATG_z1_-ATG_z2_-(CAAT)_10_-CAA ACT CC | yes  no |
| **NTHi1500 (1)** | Frame x/y: ATG_x_-ATG_y_-(CAAT)_11_-C AAA CTC C Frame z1/z2: ATG_z1_-ATG_z2_-(CAAT)_11_-CA AAC TCC | no  yes |
| **NTHi398 (2)** | Frame x/y: ATG_x_-ATG_y_-(CAAT)_12_-CAA A CT | no |
|  | Frame z1/z2: ATG_z1_-ATG_z2_-(CAAT)_12_-C AAA CT | no |
| **NTHi1553 (2)/1606 (2)/1568 (2)** | Frame x/y: ATG_x_-ATG_y_-(CAAT)_13_-CA AAC TCC  Frame z1/z2: ATG_z1_-ATG_z2_-(CAAT)_13_-CAA ACT CC | yes  no |
| **NTHi1619 (3)** | Frame x/y: ATG_x_-ATG_y_-(CAAT)_16_-CA AAC TCC | yes |
|  | Frame z1/z2: ATG_z1_-ATG_z2_-(CAAT)_16_-CAA ACT CC | no |
| **NTHi1566 (2)** | Frame x/y: ATG_x_-ATG_y_-(CAAT)_17_-C AAA CTC | no |
|  | Frame z1/z2: ATG_z1_-ATG_z2_-(CAAT)_17_-CA AAC TC | yes |
| **NTHi1556 (2)** | Frame x/y: ATG_x_-ATG_y_-(CAAT)_18_-CAA ACT C | no |
|  | Frame z1/z2: ATG_z1_-ATG_z2_-(CAAT)_18_-C AAA CTC | no |
| **NTHi1513 (2)** | Frame x/y: ATG_x_-ATG_y_-(CAAT)_19_-CT CCA TAT | no |
|  | Frame z1/z2: ATG_z1_-ATG_z2_-(CAAT)_19_-CTC CAT AT | no |
| **NTHi1623 (2)** | Frame x/y: ATG_x_-ATG_y_-(CAAT)_19_-CA AAC TCC | yes |
|  | Frame z1/z2: ATG_z1_-ATG_z2_-(CAAT)_19_-CAA ACT CC | no |
| **NTHi1549 (2)** | Frame x/y: ATG_x_-ATG_y_-(CAAT)_20_-C AAA CTC | no |
|  | Frame z1/z2: ATG_z1_-ATG_z2_-(CAAT)_20_-CA AAC TC | yes |
| **NTHi1557 (2)** | Frame x/y: ATG_x_-ATG_y_-(CAAT)_21_-CTC CAT | no |
|  | Frame z1/z2: ATG_z1_-ATG_z2_-(CAAT)_21_-CT CCA T | yes |
| **NTHi1607 (2)/1621 (2)/1622 (2)** | Frame x/y: ATG_x_-ATG_y_-(CAAT)_25_-CA AAC TCC  Frame z1/z2: ATG_z1_-ATG_z2_-(CAAT)_25_-CAA ACT CC | yes  no |
| **NTHi1558 (2)** | Frame x/y: ATG_x_-ATG_y_-(CAAT)_33_-CAA ACT C | no |
|  | Frame z1/z2: ATG_z1_-ATG_z2_-(CAAT)_33_-C AAA CTC | no |
