# Supplementary material for: Nontypable Haemophilus influenzae Displays a Prevalent Surface Structure Molecular Pattern in Clinical Isolates
Source: PLoS One. 2011 Jun 16;6(6):e21133. doi: 10.1371/journal.pone.0021133 (PMC3116884; doi:10.1371/journal.pone.0021133)
Supplement: Table S5 — P5 sequence in predicted extracellular loops 1 to 5 for representative NTHi isolates. (DOC) [file pone.0021133.s006.doc]

**Table S5.** P5 sequence in predicted extracellular loops 1 to 5 for representative NTHi isolates.

| **Loop 1** | **NTHi isolate** | **Pattern** |
| --- | --- | --- |
| **GQASFHDGINNNGAIKKD-LLGGLASYGYRRNT** | **398** | **2** |
| **GQGSFHDGINNNGAIKQH-LSSTN—YGYRRNT** | **1513** | **2** |
| **GQGSFHDGINNNGAIKED-LTLG---YGYRRNT** | **1549/1607** | **2** |
| **GQGSFHDGINNNGAIKES-LTSAS—YGYRRNT** | **1553** | **2** |
| **GQGSFHDGINNNGAIKKE-LS-TS—YGYRRNT** | **1556** | **2** |
| **GQGSFHDGINNNGAIKDA-LTSAS—YGYRRNT** | **1557** | **2** |
| **GQGSFHDGINNNGAIQEN-LTL---SYGYRRNT** | **1558** | **2** |
| **GQGSFHDGINNNGAIKGD-FSSTN—YGYRRNT** | **1559** | **1** |
| **GQGSFHDGINNNGAIKED-LSLG---YGYRRNT** | **1560** | **1** |
| **GQGSFHDGINNNGAIKGD-LSSY---YGYKRNT** | **1566** | **2** |
| **GQGSFHDGINNNGAIKKD-LSSY---YGYKRNT** | **1619** | **3** |
| **GQASFHDGINNNGAIKED-LALG---YGYRRNT** | **1621** | **2** |
| **GQGSFHDGINNNGAIKED-LSSY---YGYKRNT** | **1623** | **2** |
| **GQGSFHDGINNNGAIKQD-LGLG---YGHRRNT** | **1630** | **2** |
| **GQGSFHDGVRAMGKQFSAGHYRNT—FTYGVF-** | **1500/1606** | **1/2** |
| **GQGSFHDGINNNGAIKEN-LTSAS—YGYRRNT** | **1622** | **2** |
| **Loop 2** | **NTHi isolate** | **Pattern** |
| **DDFGRAKFRAAGKPKVKHTN** | **1630** | **2** |
| **DDFGRAKLKTTGKLTGKHTN** | **1500/1606** | **1/2** |
| **DDFGRAKFRATGKPKVKHTN** | **1558** | **2** |
| **DNFGRVKFRTEGKTTAKHTN** | **1621** | **2** |
| **DNFGRVKFRAEGKTKAKHTN** | **1549/1607** | **2/2** |
| **DNFGRVKFRAEGKAKAKHTN** | **1560** | **1** |
| **DNFGRAKLRLEGKPKAKHTN** | **398** | **2** |
| **DNFGRAKLREAGKPKVKHTN** | **1513** | **2** |
| **DNFGRAKLREVGKPSAKHTN** | **1553/1557** | **2/2** |
| **DNFGRAKLREVGDTRAKHTN** | **1556** | **2** |
| **DNFGRVKLRLAGKPKVKHTN** | **1559/1619/1623** | **1/3/2** |
| **DNFGRVKLRANGQTLAKHTN** | **1566** | **2** |
| **DNFGRAKLRLAGKPKAKHTN** | **1622** | **2** |
| **Loop 3** | **NTHi isolate** | **Pattern** |
| **VRSDYKFYEVANGTRDHKKGR** | **1553** | **2** |
| **VRSDYKFYDDANGTRDHKKGR** | **398** | **2** |
| **IRSDYKYYDQ—GVRQRAKSE** | **1500/1606** | **1/2** |
| **VRSDYKFYNDANGTRDHKKSR** | **1513** | **2** |
| **VRSDYKFYEAANGTRDHKKGR** | **1557** | **2** |
| **VRSDYKFYEEANGTRDHKKSR** | **1559/1619** | **1/3** |
| **VRSDYKFYEDANGTRDHKKGR** | **1560/1607/1621/1622/1630/1549** | **1/2/2/2/2/2** |
| **VRSDYKFYTGPNSTRDHKKGR** | **1566** | **2** |
| **VRSDYKHYEDANGTRNHDKGR** | **1556** | **2** |
| **VRSDYKFYEAANGSRDHKAGR** | **1558** | **2** |
| **VRSDYKFYEDANGTRDHKKSR** | **1623** | **2** |
| **Loop 4** | **NTHi isolate** | **Pattern** |
| **LTRVGKYRPQDK** | **398/1513/1553/1559/1622/1630/**  **1607/1623/1619/1557** | **2/2/2/1/2/2/2/2/3/2** |
| **LTRVGKYRPQAK** | **1560** | **1** |
| **LTRVGKYRTQEK** | **1566** | **2** |
| **LTRIGKYRPQAK** | **1621** | **2** |
| **VNQIGKLRSHH-** | **1500/1606** | **1/2** |
| **LTRVGKYRTQDK** | **1556** | **2** |
| **LTRVGKFRTQDK** | **1558** | **2** |
| **LTRVGKYRSQDK** | **1549** | **2** |
| **Loop 5** | **NTHi isolate** | **Pattern** |
| **RFGQGEAPVVAAPEVVSKT** | **1560** | **1** |
| **RFGQGAAPVVAAPEVVSKT** | **398/1549/1557/1559/1607/**  **1619/1630/1621/1553** | **2/2/2/1/2/3/2/2/2** |
| **RFGQGEAPVVAAPEMVSKT** | **1500/1513/1558/1556/1606/1622/**  **1623/1566** | **1/2/2/2/2/2/2/2** |
