# Supplementary material for: Nontypable Haemophilus influenzae Displays a Prevalent Surface Structure Molecular Pattern in Clinical Isolates
Source: PLoS One. 2011 Jun 16;6(6):e21133. doi: 10.1371/journal.pone.0021133 (PMC3116884; doi:10.1371/journal.pone.0021133)
Supplement: Table S6 — OapA protein sequence in the variable region identified for representative NTHi isolates. (DOC) [file pone.0021133.s007.doc]

**Table S6.** OapA protein sequence in the variable region identified for representative NTHi isolates.

| **Sequence** | **NTHi isolate** |
| --- | --- |
| **NPPAQNQMAAEQANQPESAPKAEEAANNTTAQNQSVENTPMQQNVVQAPSQMPNEMAAASVMPMPPAQAEQPQMQPVQTQAEQPKPTVPVQPMKKAVE** | **1622** |
| **NPPAQNQMAVEQANQSEFAPKAEEAANNTTAQNPLVENAPMQQNVVQSPSQMPNEMAAASVAPMQPTQAEQP------------KATAPVQPMKKAVE** | **1500/1553/1560/1549/1623/1630/1559 1513/1556/1566/1619/398** |
| **NPPAQNQMAAEQANQ--------------------PENAPMQQNVVQAPIQMPNEMAAASVAPMQPAQAEQP------------KATAPVQPMKKAVE** | **1607** |
| **NPPAQNQMAVEQANQPEFAPKAEEAANNTTAQNPLVENAPMQ-----------------------PAQAEQP------------KATAPVQPMKKAVE** | **1568** |
| **NPPAQNQM--------------------------------------------PNEMAAASVMPMQAAQAEQPQMQPAQTQAEQPKPTVPVQPMKKTVE** | **1606/1557** |
| **NPPAQNQM------------------------------------------------AAASVAPMQAAQAEQPQMQPAQTQTEQPKPTVPVQPMKKAVE** | **1558/1621** |
|  |  |
